# Study of Physico-Chemical Changes of CdTe QDs after Their Exposure to Environmental Conditions

**DOI:** 10.3390/nano10050865

**Published:** 2020-04-30

**Authors:** Bozena Hosnedlova, Michaela Vsetickova, Martina Stankova, Dagmar Uhlirova, Branislav Ruttkay-Nedecky, Augustine Ofomaja, Carlos Fernandez, Marta Kepinska, Mojmir Baron, Bach Duong Ngoc, Hoai Viet Nguyen, Ha Pham Thi Thu, Jiri Sochor, Rene Kizek

**Affiliations:** 1 Department of Viticulture and Enology, Faculty of Horticulture, Mendel University in Brno, CZ-691 44 Lednice, Czech Republic; bozena.hosnedlova@post.cz (B.H.); docekalova@preventionmedicals.cz (M.V.); brano.ruttkay@seznam.cz (B.R.-N.); mojmir.baron@mendelu.cz (M.B.); jiri.sochor@mendelu.cz (J.S.); 2 Department of Research and Development, Prevention Medicals, 742 13 Studenka-Butovice, Czech Republic; MartStan@seznam.cz (M.S.); uhlirova@preventionmedicals.cz (D.U.); 3 Department of Molecular Biology and Pharmaceutical Biotechnology, Faculty of Pharmacy, University of Veterinary and Pharmaceutical Sciences Brno, 612 42 Brno, Czech Republic; 4 Biosorption and Wastewater Treatment Research Laboratory, Department of Chemistry, Faculty of Applied and Computer Sciences, Vaal University of Technology, Vanderbijlpark 1900, South Africa; augustineo@vut.ac.za; 5 School of Pharmacy and Life Sciences, Robert Gordon University, Aberdeen AB10 7QB, UK; c.fernandez@rgu.ac.uk; 6 Department of Biomedical and Environmental Analyses, Faculty of Pharmacy, Wroclaw Medical University, 50-556 Wroclaw, Poland; marta.kepinska@umed.wroc.pl; 7 Research Center for Environmental Monitoring and Modeling, University of Science, Vietnam National University, Hanoi 100000, Vietnam; duongngocbach@hus.edu.vn (B.D.N.); nguyenviethoai@hus.edu.vn (H.V.N.); 8 Faculty of Environmental Science, University of Science, Vietnam National University, Hanoi 100000, Vietnam; thuhaee@yahoo.com; 9 Department of Human Pharmacology and Toxicology, Faculty of Pharmacy, University of Veterinary and Pharmaceutical Sciences Brno, 612 42 Brno, Czech Republic

**Keywords:** UV radiation, quantum dots, electrochemistry detection, fluorometric detection

## Abstract

The irradiance of ultraviolet (UV) radiation is a physical parameter that significantly influences biological molecules by affecting their molecular structure. The influence of UV radiation on nanoparticles has not been investigated much. In this work, the ability of cadmium telluride quantum dots (CdTe QDs) to respond to natural UV radiation was examined. The average size of the yellow QDs was 4 nm, and the sizes of green, red and orange QDs were 2 nm. Quantum yield of green CdTe QDs-MSA (mercaptosuccinic acid)-A, yellow CdTe QDs-MSA-B, orange CdTe QDs-MSA-C and red CdTe QDs-MSA-D were 23.0%, 16.0%, 18.0% and 7.0%, respectively. Green, yellow, orange and red CdTe QDs were replaced every day and exposed to daily UV radiation for 12 h for seven consecutive days in summer with UV index signal integration ranging from 1894 to 2970. The rising dose of UV radiation led to the release of cadmium ions and the change in the size of individual QDs. The shifts were evident in absorption signals (shifts of the absorbance maxima of individual CdTe QDs-MSA were in the range of 6–79 nm), sulfhydryl (SH)-group signals (after UV exposure, the largest changes in the differential signal of the SH groups were observed in the orange, green, and yellow QDs, while in red QDs, there were almost no changes), fluorescence, and electrochemical signals. Yellow, orange and green QDs showed a stronger response to UV radiation than red ones.

## 1. Introduction

Climate changes alter ecosystems, local and global weather patterns [1,2,3]. Monitoring these changes is undoubtedly very important for understanding these processes, both anthropogenic and natural [4,5]. The sun is a natural source of ultraviolet light (UV) radiation on Earth; however, only the UV-A and UV-B components of this radiation enter the troposphere. UV-A radiation has a wavelength range of 320–400 nm and represents 99% of the sun’s UV rays that fall on the Earth’s surface. UV-B radiation has a wavelength range of 280–320 nm which is largely absorbed by the ozone layer, causing direct damage to DNA and cells. UV-B radiation has been found to cause the release of Cd^2+^ ions from quantum dots (QDs) as a result of cadmium telluride (CdTe) QDs surface oxidation [6]. This mechanism can be used to construct a new, very simple, and completely non-demanding qualitative UV-B radiation sensor, even in places where technical equipment is not available. Such a sensor could be used to monitor the UV-B radiation on Earth in inaccessible areas and war zones, or also in space research, the space station, or planetary solar system research.

QDs, semiconductor nanocrystals with quantum confinement [7,8], have been increasingly used in recent years in many industrial [9,10,11,12] and biological applications [13,14,15,16,17,18]. In addition, by preparing them by green synthesis, CdTe QDs can achieve negligible toxicity and thereby increase their potential as sensors [19]. In comparison to classical staining agents, such as organic dyes and fluorescent proteins, QDs possess a high quantum yield of fluorescence, a broad excitation spectrum, and a narrow/symmetric emission spectrum [20]. Moreover, the emission spectra of several QDs can be continuously tuned by changing the particle size. Therefore, QDs with different size distribution can be excited by a single wavelength of light, making them a suitable tool for bioimaging [21,22]. Furthermore, QDs exhibit a high photobleaching threshold and excellent photostability [23]. QDs are 100 to 1000 times more stable against photobleaching and are also 10 to 100 times brighter compared to organic dyes [24]. From a physicochemical point of view, QDs are photoluminescent semiconductor nanocrystals constructed from the periodic table of elements of groups II (Zn, Cd, Hg)–VI (Se, S, and Te), III–V, and IV–VI. Until the last decade, most studies had focused on QDs of groups II–VI (most commonly CdSe or CdTe) [25,26,27]. Fisher et al. described in detail the physical characteristics of CdSe QDs [28]. A typical diameter of QDs is in the range of 1 to 20 nm and may contain between 100 and 100,000 atoms per nanoparticle [29]. Some of the most attractive QD properties include: high quantum yields, high molar extinction coefficients, wide absorption spectra, narrow and symmetric emission bands (30–50 nm), large effective Stokes shifts, and high resistance to bleaching and chemical degradation [30]. Current applications of QDs are widespread, and their use as fluorescent labels in biological tests is one of the most promising [31]. Tsipotan et al. examined the effect of visible light and UV radiation on the aggregation stability of CdTe QDs [32]. In the case of ultraviolet light, photoinduced reduction of CdTe QDs size was confirmed. Typical CdTe QDs stabilised with mercaptosuccinic acid (MSA) after exposure to specific UV-B radiation at a given wavelength and energy resulted in the excitation of a system capable of generating a whole group of molecularly reactive oxidation intermediates (ROI) and reactive oxygen species (ROS) [6,33]. Due to this photodynamic effect of CdTe QDs, free cadmium ions are released. QDs are one of the most promising nanomaterials, due to their size-dependent characteristics as well as their easily controllable size during the synthesis process [31]. Luminescent QD-semiconductor nanocrystals are a promising alternative to organic dyes for fluorescence-based applications [34]. Compared to conventional organic fluorescent dyes, QDs possess higher photoluminescence, excellent quantum yield, a size-dependent tuneable luminescence wavelength, wide continuous absorption, narrow fluorescence band, and better photostability. Over the past two decades, considerable efforts have been made to develop QD-based fluorescence probes and sensors [15,35,36,37,38]. In order to obtain an optimum quantum efficiency, the following stabilisers are commonly used: thioglycolic acid (TGA) [32,39,40], glutathione (GSH) [41], mercaptosuccinic acid (MSA) [42], 3-mercaptopropionic acid (MPA) [43] and L-cysteine [44,45]. CdTe QDs are known to possess a high fluorescence efficiency and good stability [46].

This study was aimed at studying the behavior of CdTe QDs after exposure to sunlight.

## 2. Materials and Methods

### 2.1. Chemicals and Materials

All chemicals used in this study such as Cd(CH_3_COO)_2_ · 2H_2_O, Na_2_TeO_3_, mercaptosuccinic acid (MSA), Trizma base, HCl were purchased from Sigma-Aldrich (St. Louis, MO, USA), in ACS (American chemical society grade) purity. Propanol and NaBH_4_ were purchased from Merck (Darmstadt, Germany), and a 25% aqueous ammonia solution (25% aqueous NH_3_) was purchased from Lach-Ner s.r.o. (Neratovice, Czech Republic). All chemicals that we employed for gel electrophoresis were purchased from VWR (Randor, PA, USA). All plastic materials used (tubes, tips) in this study were purchased from Eppendorf (Hamburg, Germany).

### 2.2. Deionised Water, pH, and Ion Analysis

Deionised water was prepared by using the reverse osmosis equipment Aqual 25 (Aqual s.r.o., Brno, Czech Republic), and was further purified by using an apparatus equipped with a UV lamp (ELGA, Lane End, UK). The resistance was 18 MΩ and the pH was measured using a pH meter (WTW, Berlin, Germany).

### 2.3. Synthesis of Cadmium Telluride (CdTe) Quantum Dots (QDs)

We used the procedure from our previous paper with minor modifications [47,48]. Briefly, the preparation of CdTe quantum dots (QDs) was as follows: 10 mL solution of Cd(CH_3_COO)_2_· 2H_2_O (0.02 M), 76 mL of H_2_O, 1 mL of MSA solution (0.4 M), 5 mL of Na_2_TeO_3_ (0.02 M), and 40 mg of NaBH_4_ were stirred with a magnetic stirrer (VMS-C4, VWR, Randor, PA, USA) for at least 2 h until the bubbling stopped. Subsequently, the volume was adjusted to 100 mL; 2 mL of the prepared solution was pipetted into a glass vials (Sigma Aldrich, St. Louis, MO, USA) with a white cap (Anton Paar, Graz, Austria) and a Teflon cap (Anton Paar, Graz, Austria). The vial was placed in a microwave, which was set to a power of 300 W, and the heating took place for 2 min for green QDs, 4 min for yellow QDs, 6 min for orange QDs, and 8 min for red QDs. The particles were prepared by precipitation with methanol (1:1) and left on a magnetic stirrer (60 min). After purification, the supernatant was removed and the particles were allowed to dry in a dryer DRY-Line (24 h, 60 °C, VWR, Randor, PA, USA). The final concentrations of QDs were 2 mM.

### 2.4. Absorbance Measurements

Spectrophotometry: an ultraviolet–visible (UV–Vis) UV-3100PC (VWR Randor, PA, USA) single-beam spectrophotometer was used to record the UV–Vis spectra. The Vis spectrum was measured every 2 nm in the range of 400–800 nm in plastic cuvettes with an optical path of 1 cm. An Infinite F50 (Tecan, Mannedorf, Switzerland) was used for measurement on a polystyrene microtiter plate (Gama Group a.s., Ceske Budejovice, Czech Republic). Automated spectrometric measurements: BS-300 chemical analyser from Mindray (Shenzhen, China), cuvettes 5 × 6 × 30 mm, optical path 5 mm and a volume of the reaction mixture in the cuvette 180–500 µL were used. A photometric detector measuring at wavelengths: 340, 405, 450, 510, 546, 578, 630, and 670 nm was employed. Reagents and samples were placed on the cooled sample holder (4 °C) and automatically pipetted directly into plastic cuvettes. Incubation proceeded at 37 °C. The mixture was consequently stirred. The washing steps by distilled water (18 mΩ) were done in the midst of the pipetting.

### 2.5. Fluorescence Measurements

Fluorescence spectra were obtained with the VARIOSCAN LUX (Thermo Scientific, Waltham, MA, USA). The samples were placed on a polystyrene microtiter plate (Gama Group a.s., Ceske Budejovice, Czech Republic). For fluorescence spectra, there was an excitation wavelength of 250 nm and an emission wavelength in the range 350 to 800 nm. The Vis spectrum was measured every 2 nm in the range of 350–700 nm.

### 2.6. Electrochemical Determination of Cadmium Ions

Determination of Cd^2+^ by difference pulse voltammetry (DPV) was performed at 663 VA Stand (Metrohm, Herisau, Switzerland). A standard cell with three electrodes was used, and a hanging mercury drop electrode with a drop area of 0.4 mm^2^ was employed as the working electrode. An Ag/AgCl/3M KCl electrode acted as the reference and a carbon electrode as an auxiliary. For data processing, VA database software 2.0 (Metrohm, Herisau, Switzerland) was employed. The analysed samples were deoxygenated prior to measurements by purging with argon (99.999%). Acetate buffer (0.2 M sodium acetate and 0.2 M acetic acid, pH = 5) was used as a supporting electrolyte. The parameters of the measurement were as follows: initial potential −1.2 V, end potential 0 V, deoxygenating with argon 120 s, accumulation time 120 s, step potential 5 mV, modulation amplitude 25 mV, the volume of injected sample: 50 µL and the volume of measurement cell was 10 mL. The samples, the electrolyte, and the measuring vessel were thermostated using the JULABO-200 circulation pump (Julabo GmbH, Seelbach, Germany). The temperature was set to 20 °C for all measurements.

### 2.7. Characterization of CdTe Quantum Dots by Field-Emission Scanning Electron Microscopy and High-Resolution Transmission Electron Microscopy

The nanostructure and surface morphology of the prepared CdTe QDs was characterized by field-emission scanning electron microscopy (FESEM) employing 10 kV (Zeiss, Oberkochen, Germany). Charging effect was minimized by coating the CdTe QDs samples with gold. The coated samples were then immobilized on a copper stub using carbon glue before measurements were conducted. High-resolution transmission electron microscopy (HRTEM) was conducted on a JEOL instrument (JEOL, Tokyo, Japan) to examine the surface morphology of the synthesized CdTe. The nanomaterials were mixed with absolute ethanol in vials and sonicated for 10 min. Carbon grids of 10 μm mesh size were then immersed in the solution containing the nanomaterials, dried and applied for the analysis. The determination of the individual elemental components of the CdTe QDs was performed using energy dispersive X-ray spectroscopy (EDX).

### 2.8. Zetasizer Analysis of Nanoparticles

The size distribution (i.e., the hydrodynamic diameter) was determined by dynamic light scattering (DLS) using the Zetasizer Nano ZS ZEN3600 (Malvern Instruments, Malvern, UK) with a detection angle of 173° in optically homogeneous square polystyrene cells. The samples were diluted hundredfold with deionized water. All measurements were performed at 25 °C. Each value was obtained as an average of 5 runs with at least 10 measurements. Version 7.10 of the Zetasizer Software (Malvern Instruments, Malvern, UK) was applied for data evaluation. The particle charge (ζ-potential) was measured by the microelectrophoretic method using a Malvern Zetasizer Nano ZS ZEN3600 (Malvern Instruments, Malvern, UK). All the measurements were performed at 25 °C in polycarbonate cuvettes. Each value was obtained as an average of 5 subsequent runs of the instrument with at least 20 measurements.

### 2.9. Measurement of Physical Parameters

The weather station was located on an open non-industrial landscape in Boskovice, Czech Republic. For the monitoring, two independent measuring points with the frequency parameter of 60 s were selected. We used data from the Davis meteorological station (Hayward, NJ, USA). The station is located in Boritov, with 16°58′65” E longitude and 49°43′09” N latitude. Its altitude is 305 m a.s.l. Each weather station’s temperature, humidity, and rain sensor are at 1.85 m, and solar and UV sensors are at 3.5 m. Individual parameters were collected at one-minute intervals. All data were automatically sent to the control unit and then to a computer database for data storage. The experiments were performed as follows: 1. Experiment monitoring the effect of natural radiation in one day (repeated 3 times); 2. Experiment monitoring the effect of natural radiation over 7 days (repeated 3 times).

### 2.10. Sodium Dodecyl Sulphate-Polyacrylamide Gel Electrophoresis (SDS-PAGE) for QDs Analysis

The VWR E 0322-VWR-230V electrophoretic source and the mini-horizontal electrophoretic system (VWR, Randor, PA, USA) were used for the analysis. The gels were prepared from 30% (m/V) acrylamide stock solution with 1% (m/V) bisacrylamide. The composition of the running gel was as follows: 15% (m/V) acrylamide, 0.5% (m/V) bisacrylamide, 0.1% sodium dodecyl sulphate (SDS) (m/V), 0.083% N,N,N´,N´-tetramethylethylenediamine (TEMED; V/V), 0.05% ammonium persulfate (APS; m/V) and, 0.376 M Tris/HCl at pH 8.8. The composition of the stacking gel was as follows: 4.5% acrylamide (m/V), 0.15% bisacrylamide (m/V), 0.1% SDS (m/V), 0.1% TEMED (V/V), 0.05% APS (m/V) and, 0.125 M Tris/HCl at pH 6.8. The QDs tested were diluted in a 2:1 ratio with 30% glycerol in the test tube. The gel wells were dosed with 50 μL of the thus diluted QDs. The conditions for electrophoresis were 100 V for 1.5 h in running buffer (24 mM Tris, 0.2 M glycine, and 3 mM SDS). After the electrophoresis was complete, the gel was removed and transferred to a dark room where it was illuminated by a UV lamp, and photographic documentation (Canon, Tokyo, Japan, 12 Mpx) was performed.

### 2.11. Ellman Assay

For analysis of sulfhydryl (SH) groups, Ellman spectrophotometric assay was used. 277 µL of Ellman reagent (R1) 2mM DTNB [5,5′-dithiobis(2-nitrobenzoic acid)] in 50 mM CH_3_COONa was pipetted in the cuvette, and subsequently, 45 µL of the sample was added to the mixture. 33 µL of reagent (R2) (1 M Tris CH_3_COOH, pH 8) mixture was incubated for 10 min at 37 °C. The absorbance was recorded at λ = 405 nm. The DPPH (2,2-diphenyl-1-picrylhydrazyl) method was carried out using an automated chemical analyser BS-300 (Mindray, Shenzhen, China).

### 2.12. Stability of QDs

The prepared QDs were printed on filter paper (Whatman, 1001-929, grade 1) to verify their stability. The individual samples of QDs were applied by printing (Linomat 5, Gamag, Switzerland) at a sequentially given sample volume (25 μL) from a total volume of 4 μL on the band. The print speed was 10 nL/s and, the length of the printed strip was 5 mm. After deposition of the samples, the paper was irradiated with UV light (365 nm).

### 2.13. Data Treatment and Descriptive Statistics

Experimental work was performed using at least three independent experiments. Each sample in the experiments was analysed at least five times. The obtained data presented in this paper are the average values. No experimental points were excluded from the proposed experimental study. All the obtained data were stored in the Qinslab database (Prevention Medicals, Studenka, Czech Republic). If possible, data were processed and evaluated mathematically and statistically in the Qinslab database. The results were expressed as the mean ± standard deviation (SD). Photos were processed by the ColorTest program, which assigns an intensity to the individual pixels of the studied image in a given color area.

## 3. Results

### 3.1. Characterization of CdTe Quantum Dots

The CdTe QDs were characterized by FESEM and HRTEM (Figure S1). The SEM of yellow CdTe QDs (Figure S1Aa) showed spherical particles agglomerated together with irregular sizes. The SEM analysis of green, red and orange QDs (Figure S1Ba,Ca,Da) evinced that the particles are made of irregular shaped structures of different sizes. The EDX of yellow, green and red CdTe QDs confirmed the presence of Cd and Te along with sulphur (S) and carbon (C). The EDX micrograph of orange CdTe QDs shows that the sample is basically composed of Cd, Te, S, C and trace amounts of sodium (Na). The presence of C and S in the EDX can be attributed to mercaptosuccinic acid employed in the QD synthesis. The elemental mapping for CdTe QDs exhibited that Cd and Te are spread out evenly in the composite as shown in Figure S1Ab,Bb,Cb,Db. The HRTEM for yellow CdTe QDs (Figure S1Ac) appears to show spherical groups of highly agglomerated particles. The micrograph shows the presence of lattice fringes revealing the crystalline nature of the yellow QDs. The HRTEM for green CdTe QDs (Figure S1Bc) appears to have a spherical and slightly irregular shape. The particles were only slightly agglomerated. The HRTEM for red and orange CdTe QDs (Figure S1Cc,Dc) appears to be highly irregular in shape. These particles were only slightly agglomerated. The micrograph shows the presence of lattice fringes revealing the crystalline nature of the orange QDs. The average size of the yellow, green, red and orange QDs was calculated from the histogram to be 4, 2, 2 and 2 nm, respectively.

### 3.2. Study of the Influence of Ultraviolet (UV) Radiation on Physico-Chemical Properties of QDs

Studies have been conducted to monitor the photodegradation of nanoparticles by UV radiation [49]. However, very little information is available on the photodegradation of QDs [36,50]. This is the first study to focus on the photodegradation of CdTe QDs under real conditions.

**Figure 1:**
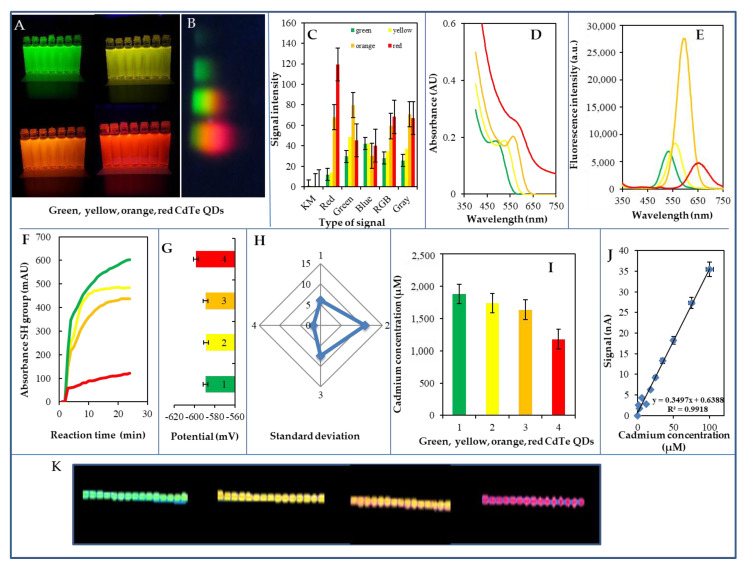
Biophysical characteristics of prepared CdTe QDs used for environmental experiment—summarizing information from all measurements. (**A**) Fluorescence of the prepared dots under ultraviolet (UV) light (310 nm) used in the experiment described above. (**B**) Typical profile of individual types of QDs on sodium dodecyl sulphate-polyacrylamide gel electrophoresis (SDS-PAGE) gel after separation (1.5 h, 100 V). (**C**) The created profile was analysed using the COLOR test program developed by us. The intensities of individual pixels are plotted in different color profiles. (**D**) Spectrophotometric characterization of QDs, plastic cuvette 1 cm track, blank solution—water. (**E**) Fluorometric characteristics of QDs measured in a UV transparent plate at excitation of 250 nm. (**F**) Typical reaction QDs curves in the presence of Ellman’s reagent for detection of free SH groups. (**G**) Changes in the peak potential of Cd in the electrochemical analysis of QDs. (**H**) Changes in standard deviation of Cd current signal during electrochemical analysis of QDs (1—green, 2—yellow, 3—orange, 4—red). (**I**) The cadmium concentration in QDs. (**J**) Typical calibration curve for cadmium analysis in organic form. (**K**) QDs deposited on paper and visualized at 365 nm the photograph made at lightening. All experiments were performed in five replicates, the displayed data being the average values. Further experimental details are described in the Materials and Methods section.

The QDs were exposed to UV radiation for 7 days. After each exposure, their basic physicochemical characteristics were evaluated. An exposure to UV-B radiation results in the photodynamic effect of MSA-stabilised CdTe QDs leading to the release of free cadmium ions. To verify the assumed hypothesis, an environmental experiment was established. Glass vials with CdTe QDs were exposed to solar and UV-B radiation for seven days at the Boritov meteorological station. As is evident from the result, significant aggregation of nanoparticles (visible change in color for all types of CdTe QDs from the original green, yellow, orange to red, and red to the dark red) occurred. We decided to use CdTe QDs stabilised with MSA in the microwave synthesis. Four types of CdTe QDs (green, yellow, orange, and red) were prepared by microwave synthesis (300 W), and their typical absorption spectra are shown in Figure 1. The determined nanoparticle size was in the range of 5 to 10 nm.

### 3.3. Biophysical Characteristics of Prepared CdTe Quantum Dots

CdTe QDs stabilised with MSA used in the study were synthesized by microwave synthesis. An assumed scheme of CdTe QDs synthesis using MPA is given in Table 1.

Four types of CdTe QD—green, yellow, orange, and red (Figure 1A)—were prepared by microwave synthesis (500 W) and under UV light (310 nm). A typical profile of the individual types of QDs on the SDS-PAGE gel after their separation is shown in Figure 1B and the intensities of the color signals of the QDs electrophoretic bands can be seen in Figure 1C. Typical absorption spectra can be seen in Figure 1D. In the visible area of the spectrum, the values for the absorption maxima were found to be as follows: green QDs 492 nm, yellow QDs 524 nm, orange QDs 565 nm, and red QDs 582 nm. Typical fluorescence maxima values are shown in Figure 1E. The fluorescence excitation/emission maxima values were as follows: green QDs 455/544 nm, yellow QDs 471/550 nm, orange QDs 535/600 nm, and red QDs 588/656 nm. Other details are shown in Table 2. The green, yellow, and orange nanoparticles had the highest number of free SH groups, while the red nanoparticles had approximately 75% fewer free SH groups (Figure 1F). The cadmium concentration was determined to be as follows: green QDs 1,878 μM, yellow QDs 1,739 μM, orange QDs 1,636 μM, and red QDs 1,181 μM (Figure 1I). The determined nanoparticle size ranged between 5 and 10 nm. The dependence of DPV signal on the Cd concentration in organic form can be seen in Figure 1J. To ascertain their stability, the prepared CdTe QDs were applied by printing to paper as a carrier, and subsequently tested for fluorescence; scanned images were evaluated by ColorTest. The prepared QDs CdTe/MSA/bio were printed (Figure 1K) on paper which sequentially was irradiated with UV light (365 nm). Within six months, a change in fluorescence of up to 10% was observed, indicating high long-term stability. The QDs stability test of QDs printed on paper for 6 months under UV light is shown in Figure 1K. The stability of the QDs thus prepared is more than 1 year (a decrease in fluorescence to a maximum of 10%).

The CdTe QDs were subsequently electrochemically determined on the working electrode. All QDs used have been found to provide very good electrochemical signals (typical green, yellow, orange, and red QDs signals). The CdTe QDs calibration relationship was linear (*r* = 0.999, LOD = 0.5 nM). Detailed information on created QDs is summarized in Table 2.

### 3.4. Biophysical Characteristics of QDs after their Exposure to UV Radiation

Four types of CdTe QDs (green, yellow, orange, and red) were exposed to UV radiation daily for 12 h (QDs were freshly prepared each day) and the measurements were repeated for seven consecutive days in summer. The summary of the results is shown in Table 3.

Table 3 gives an overview of the measured parameters for green CdTe QDs. The first two columns of the table show the integral sum of the areas of the measured solar irradiance (W/m^2^) and the UV index signal integration throughout the day. Solar radiation as well as the UV index reached the highest values on the third day and also on the fourth, sixth, and seventh days. On these days there was a shift of absorption and fluorescence maxima toward higher wavelengths. The least solar radiation and the UV index were on the first, second, and fifth days. On these days, both absorption and fluorescence maxima occurred at lower wavelengths.

The green CdTe QDs had an absorption maximum at 488 nm on the first day and at 500 nm on the third day. The absorption maximum was shifted about 12 nm toward longer wavelengths. Even greater shift of the absorption maximum occurred when comparing the second and the sixth days and the shift was about 42 nm. Similarly, in green CdTe QDs, the fluorescence peak from the first day (520 nm) was shifted about 42 nm when compared to the third day (562 nm). An even bigger shift occurred at a fluorescence peak between the first and sixth days, by 84 nm. Further, as regards the determination of cadmium concentration in the green CdTe QDs they had the highest Cd concentrations (Table 3) on the first day (1,377 μM), on the second day (1,282 μM), and on the fourth day (1,343 μM). On days with high sunlight intensities, cadmium concentrations were lower such as: on the third day (967 μM), on the sixth day (219 μM), and on the seventh day (493 μM). A similar situation was observed in SH groups, where the green CdTe QDs reached the higher absorbance (287 mAU) on the first day and lower absorbances were detected on the third (193 mAU), fifth (206 mAU) and seventh (149 mAU) day. The electrochemical potential of the green CdTe QDs reached the lowest values on the first day and on the second day (−607 mV), slightly higher values were observed from third day to seventh day (−600, −601 mV) and the highest value was obtained on the sixth day (−593 mV).

The yellow CdTe QDs had an absorption maximum at 513 nm on the first day and at 592 nm on the third day. The absorption maximum was shifted up to 79 nm toward longer wavelengths, which was the largest shift in absorption maxima for the yellow CdTe QDs. Similarly, in yellow CdTe QDs, the fluorescence peak from the first day (540 nm) was shifted about 82 nm toward longer wavelengths when compared to the third day (622 nm). An even wider shift occurred at a fluorescence peak between the first and sixth days, by 98 nm. Further, the determination of cadmium concentration in the yellow CdTe QDs showed the highest cadmium concentration on the first day (1,140 μM), on the second day (1,428 μM), and on the fifth day (1,131 μM). On days of high sunlight, the cadmium concentrations observed were lower: on the third day (414 μM), on the fourth day (561 μM), on the sixth day (371 μM), and on the seventh day (423 μM). A similar situation was observed with the SH groups, where the yellow CdTe QDs reached the higher absorbance (286 mAU) on the first day and lower absorbances were observed on the third (145 mAU), fifth (159 mAU) and seventh day (155 mAU). The electrochemical potential of the yellow CdTe QDs reached the lowest values on the second day (−606 mV), on the third day (−606 mV), slightly higher values were found on the fourth day (−603 mV), on the fifth day (−603 mV), and on the first day (−602 mV) and highest values were detected on the seventh day (−595 mV).

The orange CdTe QDs had an absorption maximum at 563 nm on the first day and at 573 nm on the third day. The absorption maximum was shifted by up to 10 nm toward longer wavelengths. An even greater shift of the absorption maximum occurs when we compare the first and sixth day by 28 nm. Similarly, the fluorescence maximum from the first day (596 nm) was shifted by 4 nm when compared to the third day (600 nm). An even wider shift occurred at a fluorescence peak between the first and sixth day by 40 nm. Furthermore, the determination of the cadmium concentration of orange CdTe QDs reached the highest cadmium concentration (1,371 μM) on the fifth day, and high cadmium concentrations were also observed on the second day (1,319 μM) and on the first day (1,150 μM). In days of high solar radiation, the orange CdTe QDs were found to have lower cadmium concentrations such as on the third day (1,122 μM), on the fourth day (1,103 μM), on the sixth day (361 μM), and on the seventh day of 582 μM. A different situation was observed with the SH groups, where the orange CdTe QDs reached higher absorbances on all days except for the sixth (146 mAU) and maximum absorbance was observed on the seventh day (309 mAU). The electrochemical potential of the CdTe QDs reached the lowest values on the second, the fourth, and the fifth days (−607 mV), slightly higher values were found on the first (−605 mV) and the third day (−604 mV). The highest values were detected on the seventh (−588 mV) and the sixth (−595 mV) days.

The red CdTe QDs had an absorption maximum at 594 nm on the first day and at 600 nm on the fourth day. The absorption maximum was shifted by 6 nm towards longer wavelengths. An even bigger displacement of the absorption maximum by 150 nm occurred when we compare the fourth and seventh days. Similarly, the fluorescence peak from the first day (654 nm) was shifted by 2 nm when compared to the third day (656 nm). An even wider shift occurred at a fluorescence peak between the second and seventh day by 14 nm. Furthermore, the cadmium concentration of orange CdTe QDs on the fifth day reached the highest value (512 μM). High cadmium concentrations were also reached on the second day (484 μM) and on the seventh day (450 μM). Cadmium concentrations of red CdTe QDs were much lower (2–3 times) in days with higher solar intensities than for green, yellow, and orange CdTe QDs (348–512 mAU). A similar situation was found with SH groups, where in red CdTe QDs absorbances were much lower (2–3 times) than in green, yellow, and orange CdTe QDs (69–88 mAU). The electrochemical potential of the red CdTe QDs reached its lowest values on the second, third, fourth, and fifth day (−607, −606 mV) slightly higher values were observed on the first (−604 mV) day and the highest values were detected on the seventh (−599 mV) and sixth (−600 mV) days.

### 3.5. Summary of Biophysical Characteristics of QDs after Their Exposure to UV Radiation

**Figure 2:**
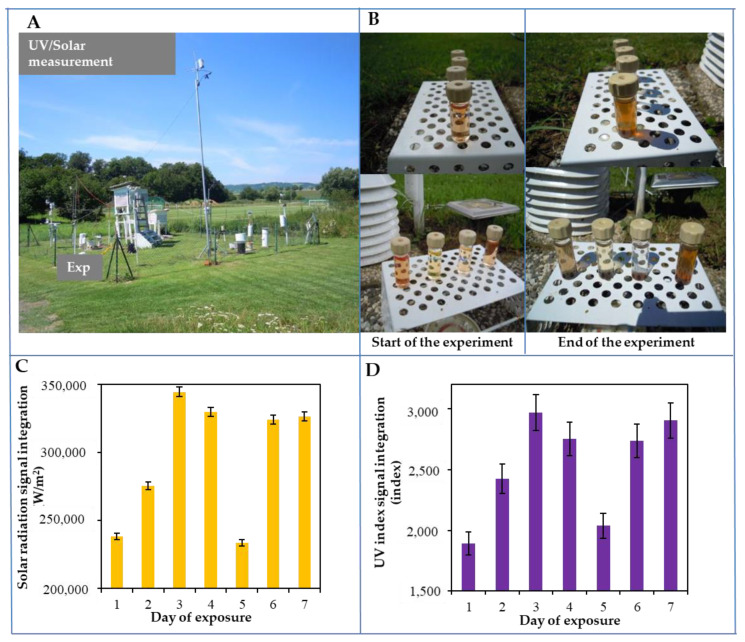
Performance of a field environmental experiment at the Boritov Biophysical Station. (**A**) The station is located in the Boskovice Trench near Maly and Velky Chlum at an altitude of 490 m a. s. l. The experiment was carried out on a plot of land with a regularly mowed common lawn (*Festuca, Lolium, Achillea, Taxaracum*). At the experiment site, the available physical data were obtained by using the Davis technique with one-minute intervals. Each test quartz vial with CdTe QDs was placed on a white platform so that nothing was shielded. A close-up of the experiment (both the beginning and the end of the experiment) is shown in part (**B**) The above figure shows the integral sum of the areas measured by both solar irradiance (W/m^2^) (**C**) and the UV index values (**D**) Further experimental details are described in the Materials and Methods section.

**Figure 3:**
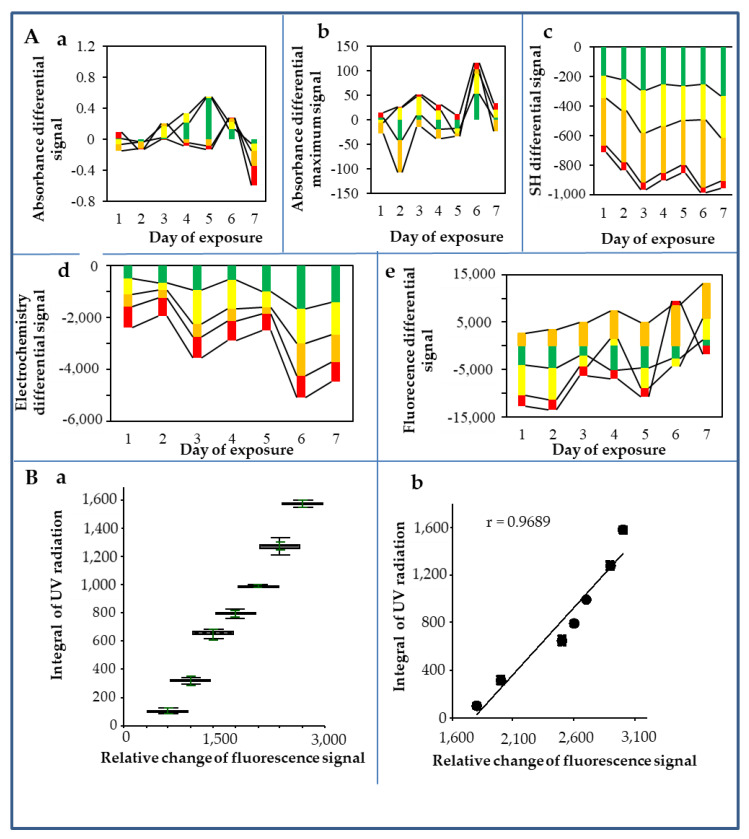
Changes in the observed biophysical parameters of CdTe QDs after exposure to the environment. (**A**) The average differential changes of CdTe QDs signals (subtracted from individual QDs without the impact of environmental exposure, i.e., controls) were: maximum absorption (**a**), peak absorption (**b**), sulfhydryl group signal (**c**), cadmium redox signal (**d**), and fluorescence signal (**e**). The individual tested color QDs are marked in the graphs with the appropriate colors. (**B**) The dependence of UV irradiance on QDs response expressed as a fluorescence signal (**a**) robust box analysis; (**b**) correlation. The results were evaluated as averages of five replicates. Further experimental details are described in the Materials and Methods section.

The integral sum of the areas of the both measured solar irradiance (W/m^2^) and the UV index is shown in Figure 2C,D. The course of the whole experiment, expressed in terms of differential changes, is shown in Figure 3 and Table 3. The highest solar and UV radiations were detected on the third, fourth, sixth, and seventh days, whereas the lowest solar and UV radiations were observed on the first and fifth days. Furthermore, differential signals were subtracted from the control QDs. The absorbance differential signal is shown in Figure 3Aa. Based on the graph, it can be concluded that the highest changes were achieved on the fourth and fifth days in the green QDs, and on the seventh day in the red and orange QDs. Similar changes can also be observed in the absorption differential signal peaks (Figure 3Ab). In the differential signal of the SH groups (Figure 3Ac), the largest changes occurred in the orange, green, and yellow QDs, while red QDs remained almost unchanged. The changes in the electrochemical signals are shown in Figure 3Ad. The signals varied mostly in orange, green, and yellow QDs. In contrast, the red QDs evinced the smallest changes. A similar trend was observed in the fluorescence differential signals, as can be seen in Figure 3Ae: the highest changes were found in yellow and green QDs, whereas the smallest changes were evident in orange and red QDs.

## 4. Discussion

Several research works have already been reported on the response of metallic QDs to UV irradiation [32,33,52,53,54]. Conversely, no illumination was observed when QDs were exposed to low-energy infrared radiation, and exposure of QDs to high-energy radiation such as X-rays leads to QD damage [52]. Some previous studies revealed that QD exposure to UV radiation releases heavy metal ions from these nanomaterials. Derfus et al. [33] reported the release of free Cd^2+^ ions from cadmium selenide (CdSe) QDs in the presence of UV radiation as a consequence of QDs surface oxidation. It was found that CdTe QDs also respond to UV radiation [6,32,52,53]. UV exposure causes the release of Cd^2+^ ions from the CdTe QD surface. It can be assumed that O_2_ molecules oxidize chalcogenide atoms located on the surface of the QDs to form oxides. These oxide molecules desorb from the surface, leaving behind “dangling” reduced Cd atoms. Therefore, prolonged exposure of QDs to an oxidative environment can lead to the decomposition of the CdTe nanocrystal, thereby leading to desorption of Cd^2+^ ions or CdTe complexes from the core QD as described in CdSe [33,55,56].

The radiation induces the aggregation of nanoparticles as observed in our study. Tsipotan et al. [32] examined the influence of irradiation by UV as well as by visible light on the photostimulated aggregation of CdTe QDs stabilised by TGA. In our study, MSA as a stabiliser of CdTe QDs was used, and a pronounced aggregation of these QDs after seven-day UV irradiation in an environment experiment was observed (Figure 2). It has been revealed that photoetching and surface-recombination processes occur during UV irradiation, which leads to improvement of the photoluminescence properties of QDs [54,57,58,59]. Photochemical interactions lead to the destruction of the stabilizer and the QDs‘ surface [32]. After exposure to an oxidative environment, a change in the color and absorbance profile of the QD solution could be observed [33,43,56]. In our laboratory experiment, UV irradiation value with a wavelength of 310 nm for 60 min was used, and the exposure resulted in a change in the color of the CdTe QDs solution (data not shown). The same finding was recorded with the exposure of QDs to a natural/atmospheric source of UV radiation in the Boritov Biophysical Station (Figure 2). After all-day of solar radiation (12 h every day for seven consecutive days), with the UV index values of 1–8 and visible light with irradiance in the range of 200–700 W/m^2^, the phenomenon of aggregation was observed. In contrast, Tsipotan et al. [32] achieved degradation of QDs’ surface up to a much higher intensity of UV radiation (0.4 W/cm^2^) and lower exposure time (75 min). Ma et al. [60,61] verified the photodetachment of TGA under 532 nm laser irradiation at 80 mW/mm^2^ (i.e., 114–400 times higher irradiance than in our study). Our findings, however, also indicate that common values of solar ultraviolet radiation provoked photochemical changes in CdTe QDs.

Besides the release of Cd^2+^ ions from CdTe QDs, it is also assumed that a surface modifier was also used—a thiol stabiliser, MSA—was oxidized, which we have confirmed in our study by measuring changes in the number of SH groups. Tsipotan et al. [32] reported that the irradiation of TGA-capped CdTe QDs by radiation with a wavelength range of 300–370 nm caused after 75 min photocatalytic oxidation and degradation of the TGA layer, i.e., under comparable conditions as in our experiment. Accompanying photooxidation of QDs leads to the reduction in their size, which, owing to the quantum confinement effect [62], results in the blue shift of the exciton absorption band. In addition, luminescence-quenching defects arise, resulting in a decrease in the luminescence intensity [32]. Tsipotan et al. [32] found that TGA illumination at 400 nm resulted in the conversion of TGA into an α-thiol-substituted acyl radical (α-TAR, S-CH_2_-CO·). They explained the drop in CdTe QDs’ luminescence under visible irradiation by the TGA detachment owing to such mechanism [32]. Likewise, thiol degradation and structural breakdown occurred in our QDs, which caused the photocatalytic changes.

Moreover, not only UV light [43] catalyses changes in QDs, but also air [63] has been reported to induce the oxidation of nanoparticle surfaces. The effect of photo-oxidation on CdTe QDs photobleaching depends on irradiation power density, oxygen concentration, and amount of QDs [60]. The higher irradiation power density and oxygen abundance and lower QDs will result in a higher photobleaching rate [60].

Derfus et al. [33] observed a progressive change in the color and absorbance profile of the QD solution after exposure to an oxidative environment—a blue-shift in the excitonic fluorescence spectra and red-shifted fluorescence peak adjacent to the excitonic fluorescence peak. The spectral shift directed toward the red area of the visible light spectrum after solar and UV exposure in CdTe QDs observed in our experiment (data not shown) is in agreement with previous oxidation studies [33,43]. The cause of the shifts of the absorption and fluorescence spectra is the decrease in the size of the nanoparticles during the oxidation process [33]. Absorbance changes with time reflect the aggregation of nanoparticle dispersions, as previous studies [64,65,66,67] have shown. As can be seen from our results (data not shown), noticeable aggregation of nanoparticles manifesting with a visible change in color in all types of CdTe QDs was observed. The change was observed from the original green, yellow, and orange color to red, and from red QDs to the dark red light.

Based on the consideration of Tsipotan et al. [32] that the phenomena observed for UV irradiated CdTe QDs solutions are generally similar to those of CdSe QDs, essentially the same mechanism leading to a change in absorption spectra can be expected for CdTe QDs. Moreover, our results are also consistent with the work of Lan et al. [54], who observed an increase in the intensity of fluorescence of ZnSe (S) QDs after UV irradiation.

A range of shifts depends on the intensity, wavelength, and exposure time of UV radiation. Nejdl et al. [42] observed dependency of changes in the fluorescence properties of CdTe QDs (fluorescence intensity and emission maximum) on the UV irradiation dose. They reported that UV radiation (with wavelengths of 254 and 312 nm) significantly changed fluorescence properties of CdTe QDs in a time range of 0–60 min. It was found that after five minutes of UV irradiation (λ = 312 nm), the fluorescence intensity increased by 37% compared to the control (without irradiation), and a change in color from green to light green was observed. Another five-minute exposure led to the increase in fluorescence intensity further by 9%, and the color turned to yellow-green. Another 10-minute irradiation caused an enhancement of fluorescence intensity by approximately 1%, and the color turned yellow. Each subsequent irradiation led to the decline in the fluorescence intensity of QDs by 10%. The last UV exposure time of 60 min resulted in a color change to orange.

In our study, the fluorescence intensity increased by increasing the UV irradiance (Table 3). The dependence of the UV irradiance on QDs response expressed as a relative change of fluorescence signal was characterized by the regression equation *y* = 0.8364*x* + 1819.6 (*r* = 0.9689). A study by Ibrahim et al. [68] demonstrated that the degree of changes in absorbance after irradiation is also affected by the composition of QDs. They studied the behavior of two biocompatible systems consisting of a core (CdSe) and core/shell (CdSe/ZnS) QDs surface modified with glutathione (GSH), exposed to photoirradiation using low laser power. Absorption spectrum of CdSe-GSH evinced its excitonic peak at 556 nm, while the peak of CdSe/ZnS-GSH was detected at 568 nm with a red shift of 12 nm. Similarly, the excitonic emission peak of CdSe-GSH was detected at 572 nm, whereas the peak of CdSe/ZnS-GSH was observed at 585 nm with a red shift of approximately 13 nm. The large red shifts observed in both the absorptive and emissive peaks of CdSe/ZnS-GSH compared to CdSe-GSH can be attributed to the strong interaction between the sulphur from the GSH ligand and the zinc of the shell coating.

In our study, the change in color as a result of the QDs’ aggregate formation was manifested in all four QDs color types (data not shown) with a shift clearly visible to the naked eye in the direction of the red part of the light spectrum (green, yellow, and orange to red, and red to dark red). Shifts in the absorption of particles within aggregates owing to electrodynamic interaction between particles were well described in previous research studies [42,47]. This interaction results in the splitting of the particles’ plasmon resonance absorption line into two components shifted to the blue and to the red with regard to the position of the isolated particle resonance [69].

The optical properties of the QDs depend on where the energy levels are. With a decreased QD size, the energy levels in the QD are further away from each other, so the light (both absorbed and emitted) has shorter wavelengths and is bluer. Conversely, with an increased QD size, the light has longer wavelengths and a redder color. The size of CdTe QDs is reduced by oxidation which leads to blue shift. At the same time, however, surface defects can be created through the oxidation process, giving rise to lower energy levels of electrons, leading to a red shift. For example, Derfus et al. [33] stated that shifts in the absorbance and fluorescence spectra occur because of a decrease in the size of the nanoparticle (loss of surface atoms due to oxidation), while the broad red-shifted fluorescence peak can be attributed to the formation of lower-energy band gaps induced by newly formed defect structures. Thus, the largest blue shift at absorbance observed in our study on the fifth day (green > orange > yellow > red QDs) and the red shift shown on the seventh day (red > orange > yellow > green QDs) (Figure 3Aa) can be explained by a much higher value of the UV signal integration on the seventh day compared to the fifth day (2,905 vs. 2,039) resulting in a higher release of Cd^2+^ compared to the fifth day (Table 3).

In our environmental experiment, we observed the tendency of continuity among the lowest number of SH groups, the lowest Cd content, and the highest fluorescence maximum, which were manifested in red QDs on all days with the exception of the sixth and seventh days when the Cd content in red QDs was not the lowest compared to three other QDs types; however, these values were low enough. The sixth and seventh days were very warm and bright sunny days, and the highest difference in electrochemistry was on the sixth day (Figure 3Ad), but the UV radiation was the highest on the second and third days. The sixth and seventh days were after the cold front and, therefore, had a dust-free atmosphere. In contrast, the third day was before the cold front, and therefore had higher air humidity and more particles in the atmosphere. The largest blue shift at absorbance was found on the fifth day: green > orange > yellow > red QDs. On the seventh day, a red shift of red > orange > yellow > green QDs was observed (Figure 3Aa).

Based on the aforementioned observations, the high drop in Cd^2+^ concentration in red CdTe QDs could be explained by the substantial aggregation of individual particles due to their oxidation and the formation of larger aggregates. Therefore, the shift was recorded towards the red area (in red QDs redder/dark red area) of the spectrum. This also explains the decrease in the number of SH groups due to their oxidation and the formation of S–S bridges. SH groups play a significant role in particle stabilisation in the moment of their oxidation, which can occur due to the formation of some oxidation molecules formed by the UV irradiation in an aqueous solution. SH groups are oxidised and more likely to form aggregates of S-S bonds. In all cases, we observed a significant decrease in the concentration of SH groups (Figure 3Ac). The change was more pronounced with the increased radiation on the third, sixth, and seventh days, especially for the orange QDs (in the following descending order: orange > yellow > green > red). On the surface of the particles, a photochemical reaction leading to MPA oxidation takes place, which results in nanocrystal aggregation and other physico-chemical changes. Changes in the destruction of QDs due to photocatalytic oxidation were also evident in the electrochemical signal corresponding to the presence of Cd^2+^ ions. Significant increases in Cd^2+^ ions content was observed on the third, sixth, and seventh days in the following descending order: yellow > orange > green > red QDs. The largest differential changes in fluorescence intensity were observed on the same days (in the following order: orange > yellow > green > red QDs).

Finally, many of our observed findings are in agreement with previous research works on the behavior of QD solutions in an electromagnetic radiation environment. In addition, we observed that the irradiation of MSA-stabilised CdTe QDs leads to detectable changes in maximum absorption and fluorescence, SH group number, and Cd^2+^ ions content, as electrochemical signals. Moreover, our study showed that CdTe QDs responds well to radiation in common atmospheric conditions.

## 5. Conclusions

This study provides an important basis for further understanding the relationship between the behavior of CdTe QDs and UV radiation. The exposure of CdTe QDs to sunlight resulted in a change in color, size and fluorescence spectra (green 520–604, yellow 540–638, orange 590–636, and red QDs: 648–662 nm). The findings show that MSA-stabilised CdTe QDs are a suitable platform for the measurement of UV irradiance. The designed CdTe QDs seems to be a promising prospect for their potential versatile use in a variety of industrial and environmental applications.

## Figures and Tables

**Table 1:** Expected scheme of synthesis of cadmium telluride quantum dots (CdTe QDs) using 3-mercaptopropionic acid (MPA = RSH).

Cd^2+^ + RSH → Cd(RS)^+^ + H^+^
TeO_2_ + 2OH^−^ → TeO_3_^2−^ + H_2_O
TeO_3_^2−^ + 4RSH → RS-Te-SR + RSSR + H_2_O + 2OH^−^
RS-Te-SR + RSH → RS-TeH + RSSR
RS-TeH + RSH → RSSR + HTe^−^ + H^+^
Cd(RS)^+^ + HTe^−^ + OH^−^ + H^+^ → CdTe(RSH) + H_2_O

Taken from the study of Shen et al. [48].

**Table 2:** Spectrometric characterization of various types of CdTe QDs.

Type of CdTe QDs	Absorbance Maximum (nm) ^a^	SD	Excitation Maximum (nm)	Emission Maximum (nm)	Difference ^b^	Color	Quantum Yield ^c^ (%)	Size of QDs (nm) ^d^
CdTe QDs-MSA-A	492 ± 2	3.6	455 ± 3	544 ± 2	−89	Green	23.0	2
CdTe QDs-MSA-B	524 ± 2	24.0	471 ± 2	550 ± 3	−79	Yellow	16.0	2
CdTe QDs-MSA-C	565 ± 2	35.8	535 ± 1	600 ± 3	−65	Orange	18.0	3
CdTe QDs-MSA-D	582 ± 2	15.9	588 ± 2	656 ± 2	−68	Red	7.0	3

^a^ Average Vis spectra (300–700 nm), *n* = 3; ^b^ The difference was calculated as follows: Excitation—Emission; ^c^ Quantum yield was determined according to Sousa et al. [51]; ^d^ The size was determined by transmission electron microscope (TEM) analysis.

**Table 3:** Measured parameters of green, yellow, orange and red CdTe QDs exposed to outdoor UV radiation for seven consecutive days in summer.

Day	Solar Radiation ^a^	UV Index ^b^	Vis Absorption Maximum ^c^	Fluorescence Maximum ^d^	DPV Signal ^e^	Ellman Reaction ^f^	Potential
	(W/m^2^)		(nm)	(nm)	(µM)	(mAU)	(mV)
Green							
1	238,456	1,894	488 ± 2	520 ± 2	1,377 ± 48	287 ± 14	−607
2	275,719	2,428	481 ± 2	526 ± 2	1,282 ± 56	267 ± 11	−607
3	344,679	2,970	500 ± 2	562 ± 2	967 ± 36	193 ± 14	−600
4	329,919	2,753	484 ± 2	540 ± 2	1,343 ± 56	232 ± 14	−601
5	233,861	2,039	490 ± 2	540 ± 2	891 ± 19	206 ± 13	−601
6	324,228	2,738	523 ± 2	604 ± 2	219 ± 14	232 ± 11	−593
7	326,704	2,905	530 ± 2	590 ± 2	493 ± 22	149 ± 8	−600
Yellow							
1	238,456	1,894	513 ± 2	540 ± 2	1,140 ± 66	286 ± 11	−602
2	275,719	2,428	521 ± 2	562 ± 2	1,428 ± 55	228 ± 13	−606
3	344,679	2,970	592 ± 2	622 ± 2	414 ± 22	145 ± 11	−606
4	329,919	2,753	572 ± 2	620 ± 2	561 ± 36	159 ± 14	−603
5	233,861	2,039	540 ± 2	580 ± 2	1,131 ± 22	205 ± 16	−603
6	324,228	2,738	550 ± 2	638 ± 2	371 ± 29	199 ± 13	−599
7	326,704	2,905	560 ± 2	614 ± 2	423 ± 19	155 ± 10	−595
Orange							
1	238,456	1,894	563 ± 2	596 ± 2	1,150 ± 26	285 ± 16	−605
2	275,719	2,428	566 ± 2	598 ± 2	1,319 ± 23	251 ± 13	−607
3	344,679	2,970	573 ± 2	600 ± 2	1,122 ± 21	269 ± 13	−604
4	329,919	2,753	563 ± 2	594 ± 2	1,103 ± 28	286 ± 12	−607
5	233,861	2,039	571 ± 2	594 ± 2	1,371 ± 22	300 ± 14	−607
6	324,228	2,738	591 ± 2	636 ± 2	361 ± 8	146 ± 9	−595
7	326,704	2,905	567 ± 2	590 ± 2	582 ± 9	309 ± 11	−588
Red							
1	238,456	1,894	594 ± 2	654 ± 2	402 ± 5	78 ± 2	−604
2	275,719	2,428	598 ± 2	648 ± 2	484 ± 5	70 ± 2	−607
3	344,679	2,970	587 ± 2	656 ± 2	385 ± 7	72 ± 3	−606
4	329,919	2,753	600 ± 2	650 ± 2	426 ± 8	73 ± 2	−606
5	233,861	2,039	595 ± 2	654 ± 2	512 ± 6	69 ± 1	−606
6	324,228	2,738	592 ± 2	656 ± 2	348 ± 6	88 ± 2	−600
7	326,704	2,905	564 ± 2	662 ± 2	450 ± 5	73 ± 3	−599

^a^ The daily integral of total radiation measured by the solarimeter at 3 m above the ground (W/m^2^); ^b^ The daily integral of total UV radiation index at 3 m above the ground; ^c^ The maximal value determined from the measured Vis spectrum.; ^d^ The maximal value determined from the measured fluorescence spectrum; **^e^** Concentration of cadmium ions present in the observed solution determined by DPV; **^f^** Ellman reaction—signal from free –SH (sulfhydryl) groups bound on quantum dots. *n*—number of measurements (*n* = 3).

## Supplementary Materials

The following are available online at https://www.mdpi.com/2079-4991/10/5/865/s1: Figure S1: Scanning electron microscopy (SEM) (a), energy dispersive X-ray spectroscopy (EDX) elemental mapping (b) and high-resolution transmission electron microscopy (HRTEM) (c) of CdTe quantum dots (QDs). (A) Yellow, (B) green, (C) red and (D) orange CdTe QDs. Further experimental details are described in the Materials and Methods section.

## Abbreviations

CdSe CdTe	cadmium selenide cadmium telluride
DLS DNTB DPPH DPV EDX FESEM GSH HRTEM LOD	dynamic light scattering 5,5′-dithiobis(2-nitrobenzoic acid) 2,2-diphenyl-1-picrylhydrazyl differential voltammetry energy-dispersive X-ray spectroscopy field emission scanning electron microscopy glutathione high resolution transmission electron microscopy limit of detection
LOQ	limit of quantification
MPA MSA NPs	3-mercaptopropionic acid mercaptosuccinic acid nanoparticles
P QDs	*p*-value quantum dots
*r*	correlation coefficient
ROI ROS RSD SD SDS-PAGE gel SEM	reactive oxidation intermediates reactive oxygen species relative standard deviation standard deviation sodium dodecyl sulfate-polyacrylamide gel scanning electron microscopy
SH groups SS bonds TEM TGA UV radiation	sulfhydryl groups disulfide bonds transmission electron microscopy thioglycolic acid ultraviolet radiation
