# Supplementary material for: Study of Physico-Chemical Changes of CdTe QDs after Their Exposure to Environmental Conditions
Source: Nanomaterials (Basel). 2020 Apr 30;10(5):865. doi: 10.3390/nano10050865 (PMC7279304; doi:10.3390/nano10050865)
Supplement: Supplementary file 1 [file nanomaterials-10-00865-s001.pdf]

Supplement

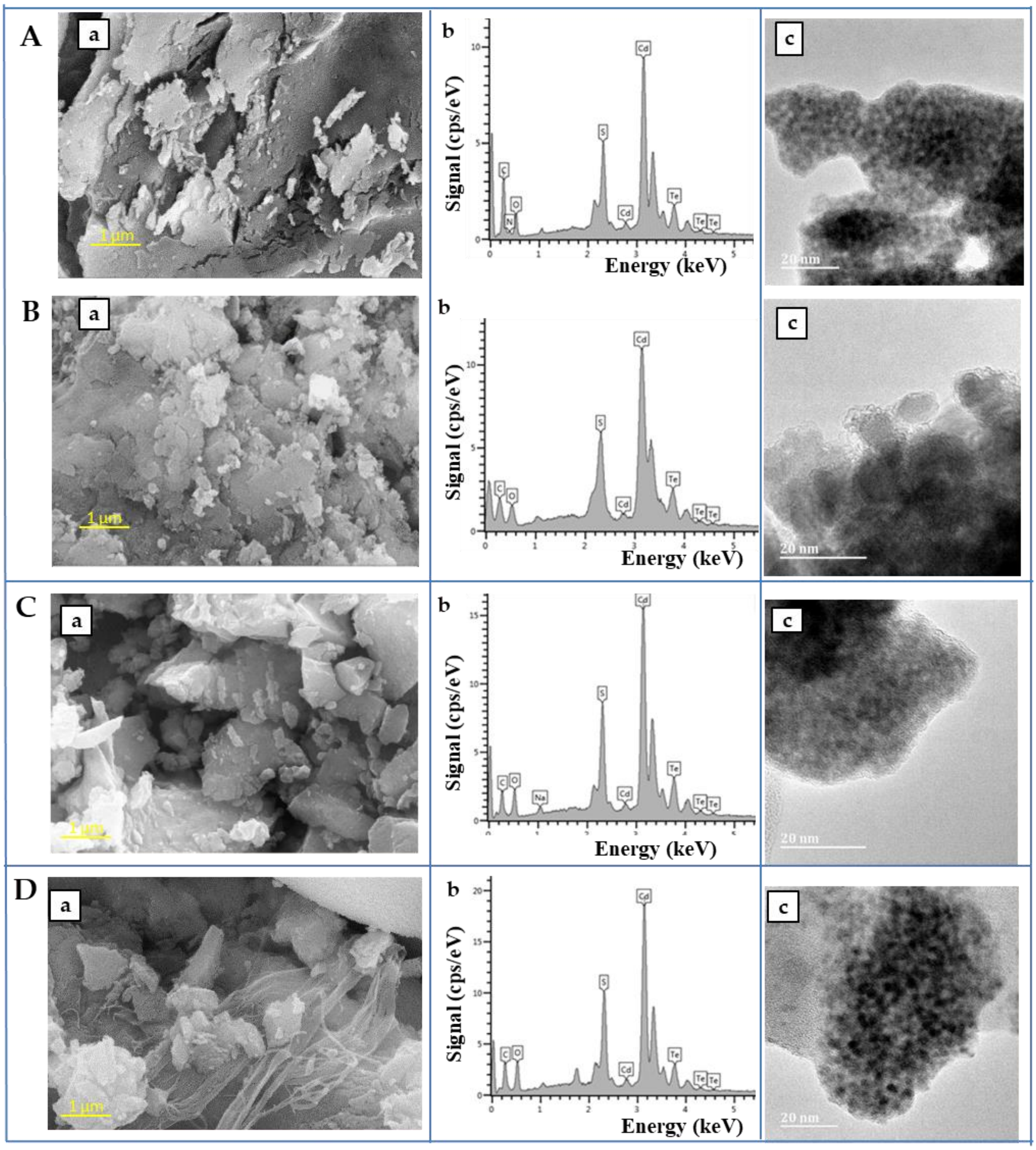

Figure S1. SEM (**a**), EDX elemental mapping (**b**) and HRTEM (**c**) of CdTe quantum dots (QDs). (**A**) Yellow, (**B**) green, (**C**) red and (**D**) orange CdTe QDs. Further experimental details are described in the Materials and Methods section.
